# The Practice Market

**Published:** 1911-03-25

**Authors:** 


### SPECIAL ARTICLES.

### THE PRACTICE MARKET.

### II.—SELLING A PRACTICE.

In our introductory article we laid stress upon the fact that every general practice is a business concern, which—at least from the medical agent's point of view—has a definite market value. To the agent a practice is simply a marketable commodity to be disposed of either wholly or in part, along certain well established lines. Its market value varies according to the terms of partnership or succession proposed by the vendor, and is mainly based, for the purposes of transfer, upon an average of the gross annual cash receipts for the preceding three years. Medical agents work largely by rule of thumb in these matters; the prospective vendor, usually after a personal interview, furnishes as best he can the answers to a stereotyped form of inquiry: and an expert estimate of the cash value of the practice or share is often available by return of post.

The agent's scheduled list of questions—cold, callous, and mercilessly inquisitive—often gives a rude shock to the easy-going practitioner of sixty, who had thought to round off his professional activities with a good bargain which will comfort and sustain him in his retirement. It is often only when he tries to dispose of the goodwill of his practice, or to take an active, congenial, and efficient partner upon favourable terms, that the long-established family doctor realises how much his real lack of business habits in the past has prejudiced his position in the present. He may have supposed that, with the aid of a dispenser or a devoted wife, the business side of his practice has been run on business lines. His conscience tells him that his accounts have been kept with scrupulous honesty—often indeed with surpassing generosity—towards his patients. Yet, under the frigid cross-examination of the agent's form of inquiry, he now sees only too clearly that in the suspicious eyes of a purchaser his practice is not what he had thought it to be.

This article will have served its purpose if it awakens the average general practitioner, of average unbusinesslike habits, to the vast importance, to himself and his family, of so conducting the financial side of his work that, in the event of his death or disablement or retirement, or of his taking in a partner, his practice may not appear upon the agent's books in its most unfavourable light.

### Keeping Accounts.

Every medical man in general practice should make at the end of each year a balance sheet showing accurately his gross receipts, and his professional expenses of every kind. The latter should be grouped under their proper headings, such as locomotion; surgery (rent and rates); instruments, dressings, appliances; locum tenens' or assistants' salaries; drugs, bottles and dispenser's salary or chemist's bill; book-keeping, etc.

Wherever possible the vendor's reason for giving up practice or taking in a partner should be stated with the utmost candour; and if possible any marked decline or increase in the receipts should be accounted for. The purchaser has his capital and his career at stake, and we believe that it is in the best interests of a *bonâ fide* vendor of a sound opening to err on the side of frankness, even in the preliminary particulars of his practice which he furnishes in the first instance to the agent. If he is too reticent at the outset he may suffer for it by being brought into communication with a succession of wholly unsuitable candidates, who will waste his time and perhaps precipitate him into a most unsatisfactory bargain.

### Methods of Sale.

So far we have only spoken of selling practices through an agent. Of course there are other methods, and indeed it may safely be said that many of the very best practices and partnerships are disposed of privately or semi-privately. Nevertheless, the large majority of medical men, wishing to transfer the whole or a share of their practices, enter the offices of one or more of the better known medical agencies.

Broadly speaking, there are five methods of selling a practice or taking in a partner: (1) Privately, through friends or relations, which includes the introduction of sons or nephews into family practices; (2) semi-privately through friends or acquaintances on the senior staff of the vendor's hospital and medical school; (3) through advertisements in the medical press; (4) through the special agency in connection with a large medical school; and (5) through the ordinary medical agencies.

### The Method of Choice.

The first two methods are usually by far the most satisfactory; but such means are not open to all medical men for a variety of reasons. Where possible they should be the vendor's "first string." Advertisements in medical journals are as often as not the refuge of the destitute or the means adopted at the outset by shady and unscrupulous practitioners who fear the cold searchlight of a respectable agency. The last two methods have this objection in the eyes of the vendor, that in spite of the expert advice which they offer and the wide field they open out for an early sale, they involve him in the payment of commission. A common fee charged by agents to the principal is 5 per cent. upon the first £500 paid by the purchaser and 2½ per cent. upon all further payments. This, of course, is a heavy tax upon the vendor.

### Hospital Agencies.

The advantage of a private hospital agency is that the whole transaction is often more personal and reliable than in the case of the larger firms. Both vendor and purchaser are usually well known to the agent and often to each other, and it is especially important to the agent to please both parties.

When dealing with an agency, the vendor may feel satisfied that his side of the bargain will not be neglected, for obvious reasons: the agent acts on commission for the vendor, and endeavours to find him a satisfactory purchaser upon the best possible terms. He sends out preliminary typewritten particulars of the opening to all the candidates upon his books who seem at all suitable; and to those who feel disposed to negotiate he supplies, under certain obvious restrictions, the name and address of the vendor, which have hitherto been suppressed.

Here we would suggest that the vendor should seldom hesitate to give an interview to a prospective purchaser, or to treat him with the utmost candour and cordiality, however unlikely it may seem at first that they will come to terms.

A sound practice with an introduction of six months is usually sold at about one and a half year's purchase, based (as always) upon an average of the three previous years' gross receipts.

*(To be continued.)*

